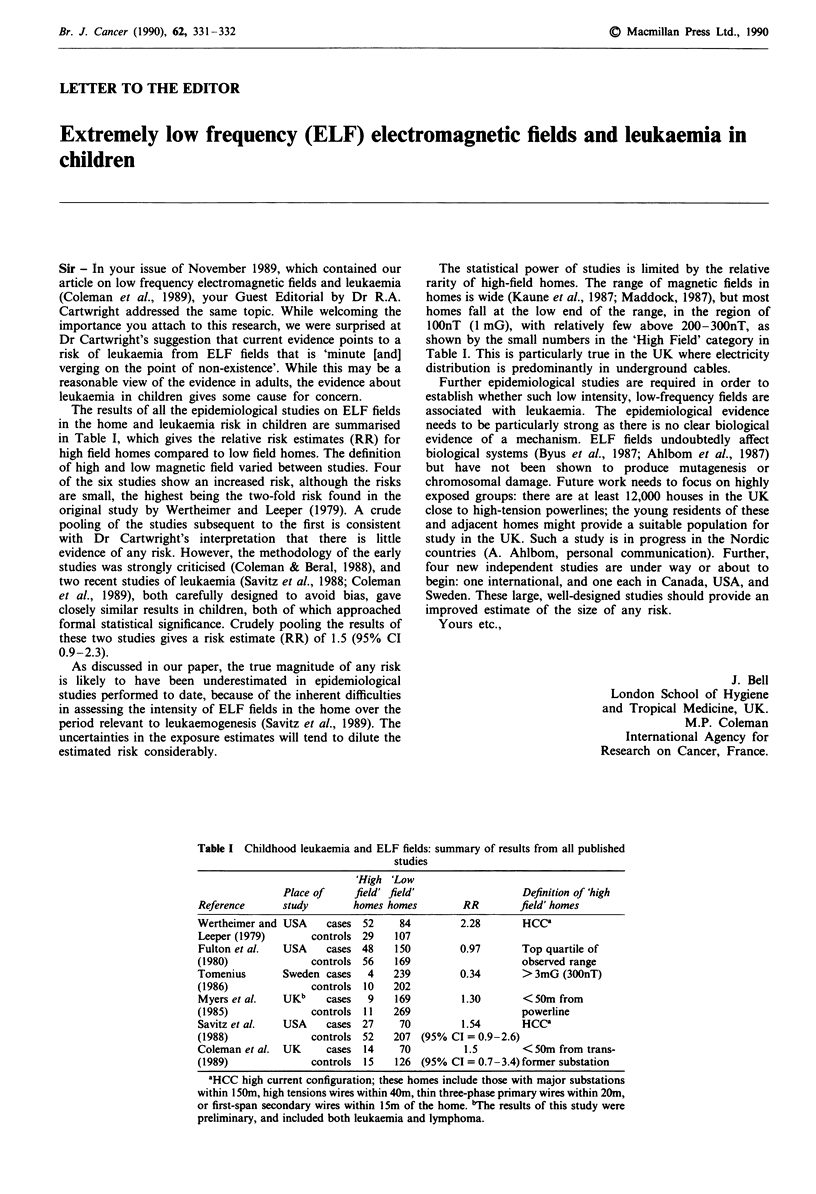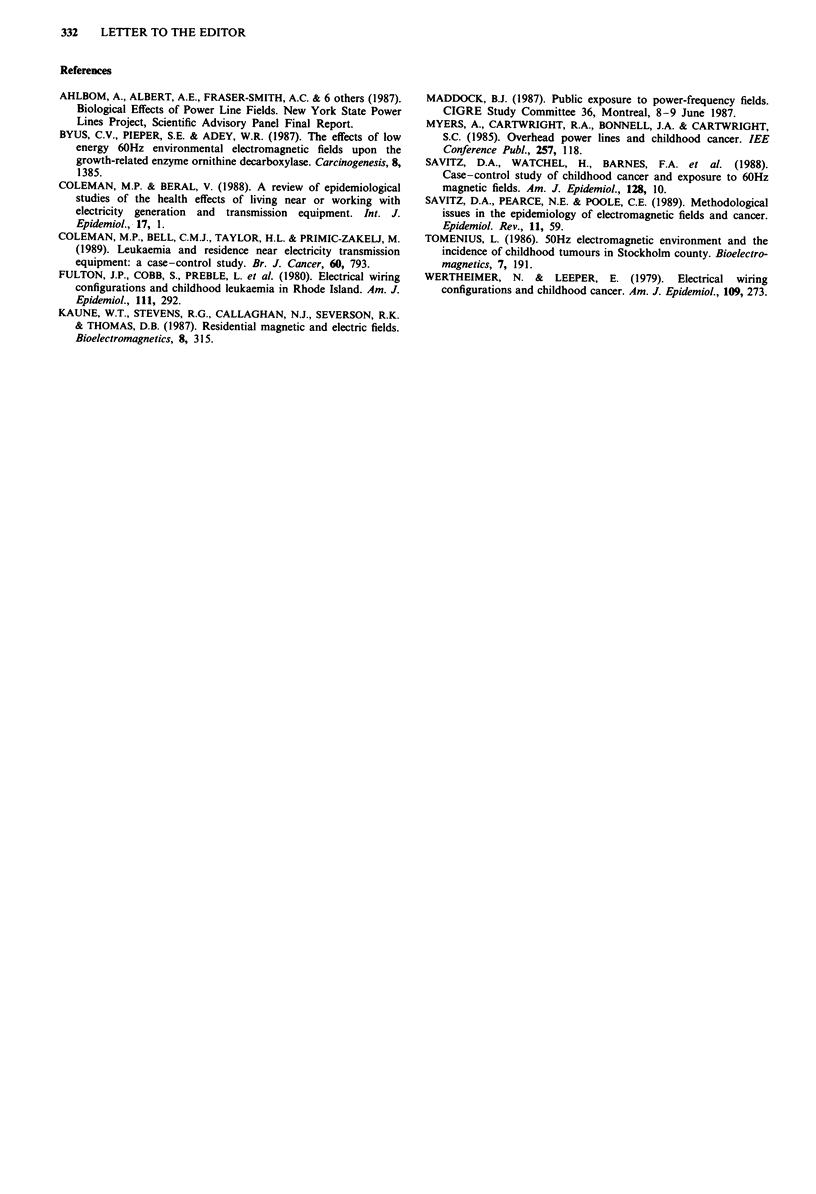# Extremely low frequency (ELF) electromagnetic fields and leukaemia in children.

**DOI:** 10.1038/bjc.1990.289

**Published:** 1990-08

**Authors:** J. Bell, M. P. Coleman


Br. J. Cancer (1990), 62, 331-332 © Macmillan Press Ltd., 1990

LETTER TO THE EDITOR

### Extremely low frequency (ELF) electromagnetic fields and leukaemia in children

Sir – In your issue of November 1989, which contained our article on low frequency electromagnetic fields and leukaemia (Coleman *et al.*, 1989), your Guest Editorial by Dr R.A. Cartwright addressed the same topic. While welcoming the importance you attach to this research, we were surprised at Dr Cartwright's suggestion that current evidence points to a risk of leukaemia from ELF fields that is 'minute [and] verging on the point of non-existence'. While this may be a reasonable view of the evidence in adults, the evidence about leukaemia in children gives some cause for concern.

The results of all the epidemiological studies on ELF fields in the home and leukaemia risk in children are summarised in Table I, which gives the relative risk estimates (RR) for high field homes compared to low field homes. The definition of high and low magnetic field varied between studies. Four of the six studies show an increased risk, although the risks are small, the highest being the two-fold risk found in the original study by Wertheimer and Leeper (1979). A crude pooling of the studies subsequent to the first is consistent with Dr Cartwright's interpretation that there is little evidence of any risk. However, the methodology of the early studies was strongly criticised (Coleman & Beral, 1988), and two recent studies of leukaemia (Savitz *et al.*, 1988; Coleman *et al.*, 1989), both carefully designed to avoid bias, gave closely similar results in children, both of which approached formal statistical significance. Crudely pooling the results of these two studies gives a risk estimate (RR) of 1.5 (95% CI 0.9-2.3).

As discussed in our paper, the true magnitude of any risk is likely to have been underestimated in epidemiological studies performed to date, because of the inherent difficulties in assessing the intensity of ELF fields in the home over the period relevant to leukaemogenesis (Savitz *et al.*, 1989). The uncertainties in the exposure estimates will tend to dilute the estimated risk considerably.

The statistical power of studies is limited by the relative rarity of high-field homes. The range of magnetic fields in homes is wide (Kaune *et al.*, 1987; Maddock, 1987), but most homes fall at the low end of the range, in the region of 100nT (1 mG), with relatively few above 200-300nT, as shown by the small numbers in the 'High Field' category in Table I. This is particularly true in the UK where electricity distribution is predominantly in underground cables.

Further epidemiological studies are required in order to establish whether such low intensity, low-frequency fields are associated with leukaemia. The epidemiological evidence needs to be particularly strong as there is no clear biological evidence of a mechanism. ELF fields undoubtedly affect biological systems (Byus *et al.*, 1987; Ahlbom *et al.*, 1987) but have not been shown to produce mutagenesis or chromosomal damage. Future work needs to focus on highly exposed groups: there are at least 12,000 houses in the UK close to high-tension powerlines; the young residents of these and adjacent homes might provide a suitable population for study in the UK. Such a study is in progress in the Nordic countries (A. Ahlbom, personal communication). Further, four new independent studies are under way or about to begin: one international, and one each in Canada, USA, and Sweden. These large, well-designed studies should provide an improved estimate of the size of any risk.

Yours etc.,

J. Bell
London School of Hygiene
and Tropical Medicine, UK.
M.P. Coleman
International Agency for
Research on Cancer, France.

**Table I** Childhood leukaemia and ELF fields: summary of results from all published studies

| *Reference* | *Place of study* | | *'High field' homes* | *'Low field' homes* | *RR* | *Definition of 'high field' homes* |
|---|---|---|---|---|---|---|
| Wertheimer and | USA | cases | 52 | 84 | 2.28 | HCC[a] |
| Leeper (1979) | | controls | 29 | 107 | | |
| Fulton *et al.* | USA | cases | 48 | 150 | 0.97 | Top quartile of |
| (1980) | | controls | 56 | 169 | | observed range |
| Tomenius | Sweden | cases | 4 | 239 | 0.34 | >3mG (300nT) |
| (1986) | | controls | 10 | 202 | | |
| Myers *et al.* | UK[b] | cases | 9 | 169 | 1.30 | <50m from |
| (1985) | | controls | 11 | 269 | | powerline |
| Savitz *et al.* | USA | cases | 27 | 70 | 1.54 | HCC[a] |
| (1988) | | controls | 52 | 207 | (95% CI = 0.9-2.6) | |
| Coleman *et al.* | UK | cases | 14 | 70 | 1.5 | <50m from trans- |
| (1989) | | controls | 15 | 126 | (95% CI = 0.7-3.4) | former substation |

[a]HCC high current configuration; these homes include those with major substations within 150m, high tensions wires within 40m, thin three-phase primary wires within 20m, or first-span secondary wires within 15m of the home. [b]The results of this study were preliminary, and included both leukaemia and lymphoma.

### References

AHLBOM, A., ALBERT, A.E., FRASER-SMITH, A.C. & 6 others (1987). Biological Effects of Power Line Fields. New York State Power Lines Project, Scientific Advisory Panel Final Report.

BYUS, C.V., PIEPER, S.E. & ADEY, W.R. (1987). The effects of low energy 60Hz environmental electromagnetic fields upon the growth-related enzyme ornithine decarboxylase. *Carcinogenesis*, **8**, 1385.

COLEMAN, M.P. & BERAL, V. (1988). A review of epidemiological studies of the health effects of living near or working with electricity generation and transmission equipment. *Int. J. Epidemiol.*, **17**, 1.

COLEMAN, M.P., BELL, C.M.J., TAYLOR, H.L. & PRIMIC-ZAKELJ, M. (1989). Leukaemia and residence near electricity transmission equipment: a case-control study. *Br. J. Cancer*, **60**, 793.

FULTON, J.P., COBB, S., PREBLE, L. *et al.* (1980). Electrical wiring configurations and childhood leukaemia in Rhode Island. *Am. J. Epidemiol.*, **111**, 292.

KAUNE, W.T., STEVENS, R.G., CALLAGHAN, N.J., SEVERSON, R.K. & THOMAS, D.B. (1987). Residential magnetic and electric fields. *Bioelectromagnetics*, **8**, 315.

MADDOCK, B.J. (1987). Public exposure to power-frequency fields. CIGRE Study Committee 36, Montreal, 8–9 June 1987.

MYERS, A., CARTWRIGHT, R.A., BONNELL, J.A. & CARTWRIGHT, S.C. (1985). Overhead power lines and childhood cancer. *IEE Conference Publ.*, **257**, 118.

SAVITZ, D.A., WATCHEL, H., BARNES, F.A. *et al.* (1988). Case–control study of childhood cancer and exposure to 60Hz magnetic fields. *Am. J. Epidemiol.*, **128**, 10.

SAVITZ, D.A., PEARCE, N.E. & POOLE, C.E. (1989). Methodological issues in the epidemiology of electromagnetic fields and cancer. *Epidemiol. Rev.*, **11**, 59.

TOMENIUS, L. (1986). 50Hz electromagnetic environment and the incidence of childhood tumours in Stockholm county. *Bioelectromagnetics*, **7**, 191.

WERTHEIMER, N. & LEEPER, E. (1979). Electrical wiring configurations and childhood cancer. *Am. J. Epidemiol.*, **109**, 273.